# Utility of sodium tetradecyl sulfate sclerotherapy from benign oral vascular lesion

**DOI:** 10.1186/s40902-016-0094-9

**Published:** 2016-11-25

**Authors:** Bo-Eun Choi, Yongsoo Kim, Dae-Ho Leem, Jin-A Baek, Seung-O Ko

**Affiliations:** Department of Oral & Maxillofacial Surgery, School of Dentistry and Institute of Oral Bioscience, Chonbuk National University, 567, Baekje-daero, Deokjin-gu, Jeonju-si, Jeollabuk-do 54896 South Korea

**Keywords:** Sclerotherapy, Hemangioma, Vascular malformation, Sodium tetradecyl sulfate

## Abstract

**Background:**

Hemangioma and vascular malformation are benign vascular lesions that often occur in cephalic and cervical region. Currently, surgical resection, laser therapy, angiographic embolization, use of steroids, and sclerotherapy are used as treatments.

**Case presentation:**

This study reports three cases of benign vascular lesions that are remarkably treated by sodium tetradecyl sulfate (STS) injection, of which occurred in oral cavity and around the mouth. Three percent of STS was diluted with 0.9 % of normal saline, and it was injected to the lesion site at least once. The result of treatment was evaluated based on clinical findings.

**Conclusion:**

Surgical treatment of hemangioma and vascular malformation occurred in oral cavity is not normally used because of esthetic issues and potential hemorrhage. On the other hand, sclerotherapy using STS is an effective therapy compare to surgical treatment. Despite the number of STS injection was different for each patient, all three patients had reached satisfactory level through the treatment with gradual diminution of lesions.

## Background

Hemangioma and vascular malformation are common benign lesions of vessel that often occur in cephalic and cervical regions. The comprehensive term “hemangioma” was classified into two (i.e., hemangioma and vascular malformation) by Mulliken and Glowacki in 1982 [1].

Hemangiomas are endothelial tumors that exhibit endothelial proliferation with rapid growth and gradual regression. They are classified into superficial, deep, and compound hemangioma according to the depth of the lesion. These lesions are more common in females. Craniofacial region is the most affected site (60 %) [2]. Hemangioma in oral cavity may include pain, hemorrhage, secondary infection, ulcerative lesion, and tissue transformation [3–5].

Vascular malformations are structural abnormalities without endothelial cell proliferation. Vascular malformations can be sub-divided into two types (i.e., low and high flow) due to hemodynamic features [6]. Low-flow vascular malformations include capillary, venous, and lymphatic lesions depending on the type of vessels. High-flow vascular malformations include arterial malformation [2]. The most common regions where it occurs in the oral cavity are two thirds of the anterior tongue, palate, gingiva, and buccal mucosa [7]. It generally appears at birth and increases its size as it grows. Functional difficulty may occur such as swallowing, maintaining the respiratory tract, and obstructive sleep apnea when vascular lesion involves deep tissue such as muscle and bone [8]. The lesions on the face cause esthetic problems, even during regression state.

Various methods for the treatment of hemangioma and vascular malformations have been introduced. There are continuous observation, radiation therapy, laser therapy, steroid treatment, sclerotherapy, and surgical resection.

We report three cases of benign vascular lesions that are remarkably treated by sodium tetradecyl sulfate (STS) injection which occurred in oral cavity esthetically and functionally.

## Case presentation

### Case 1

**Fig. 1:**
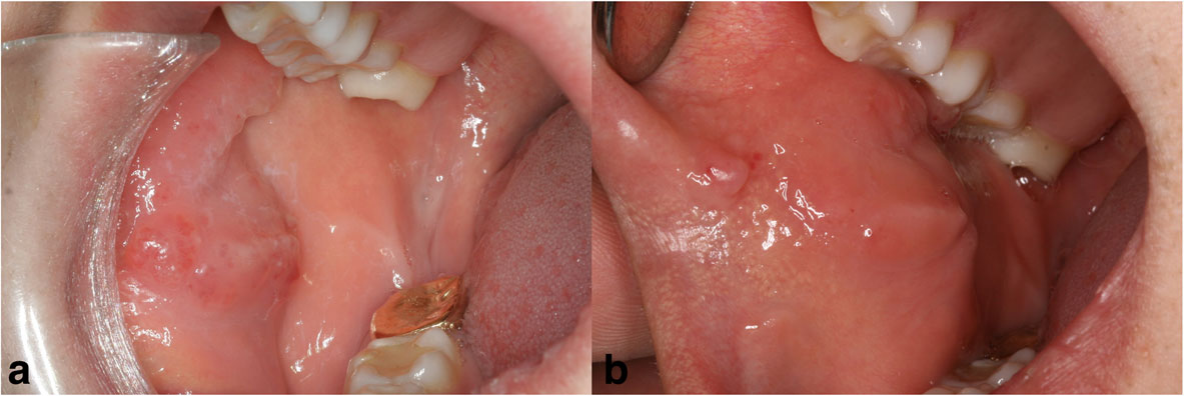
**a** Cavernous hemangioma on the right buccal mucosa. **b** Three-month postoperative appearance

A 27-year-old woman with chief complaint of repetitive swelling and bruise on the right buccal mucosa visited the maxillofacial department. The patient has recognized the lesion for about 4 years. During oral examination, a painless bluish red lesion with the size of 3 × 2 × 0.5 cm was observed at the right buccal mucosa. Bluish dome-shaped lesion was located at the center and red region scattered around the buccal mucosa (Fig. 1a). On extraoral examination, slight facial swelling was observed. The patient complained tingling sensation rather than pain. Panoramic radiograph result was nothing special. T2-weighted magnetic resonance imaging presented mass lesion with ill-defined margin in the right lower subcutaneous area. Incisional biopsy revealed that the lesion was cavernous hemangioma. 1.5 cm^3^ of 1 % STS was injected into the lesion two times. When the first injection was administered, the patient complained about pain and burning sensation on the right buccal area. After 3 weeks of the first procedure, the size of the lesion decreased to 2 × 1.5 × 0.3 cm. After the second procedure was carried out, the size of the lesion had not altered yet the thickness was reduced. After 2 months of the second procedure, 1.5 cm^3^ of 1 % STS was injected to the remaining lesion. Comparing from the first visit, the size of the lesion was regressed to 0.5-cm nodule (Fig. 1b).

### Case 2

**Fig. 2:**
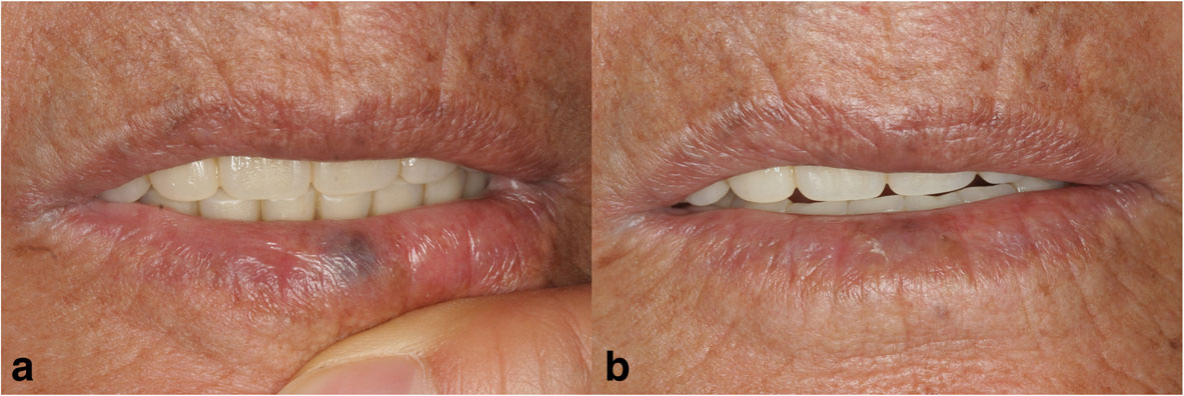
**a** Localized dome-shaped swelling in the midline of the lower lip. **b** After a week, the color became similar to adjacent tissue

A 69-year-old woman with bluish circular lesion on the lower lip visited the department. The patient had neither history of pain nor ulceration on the area. A localized dome-shaped swelling was detected in the midline of the lower lip. The patient did not complain any pain on palpation, and the size was approximately 0.6 × 0.6 × 0.1 cm (Fig. 2a). The patient has recognized the lesion for about 15 years and had no plan to visit the clinics since the lesion had no symptoms. Based on the clinical finding, provisional diagnosis was venous malformation. 0.2 cm^3^ of 1 % STS was injected, and after a week, the lesions faded and the color became similar to adjacent tissue (Fig. 2b).

### Case 3

**Fig. 3:**
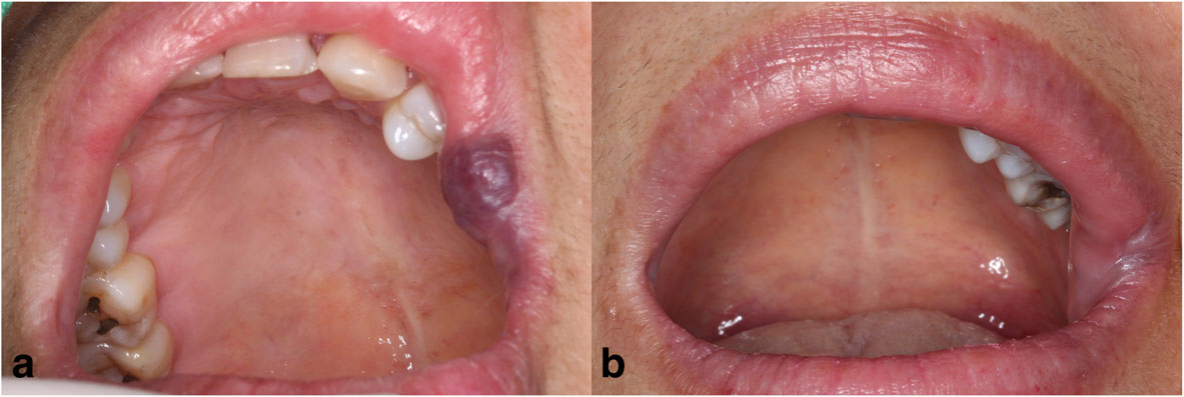
**a** Well-defined border and dome-shaped swelling on the left upper lip. The patient was diagnosed with venous malformation. **b** After 3 weeks later, the affected site almost healed

A 62-year-old female with chief complain of a bluish dome-shaped swelling on the left upper lip visited the department. The patient has recognized the lesion for about 4 years. The patient had neither history of pain nor ulceration. Border of the lesion was well defined and the size was approximately 0.8 × 0.8 × 0.3 cm (Fig. 3a). The patient was clinically diagnosed with venous malformation. By using sclerotherapy, 1.0 cm^3^ of 1 % STS was given at the site. Regression of intraoral venous malformation was observed after 3 weeks. The affected sites almost healed and got flatten (Fig. 3b).

### Discussion

Hemangioma is a benign neoplasm which blood vessels grow abnormally. Hemangioma, according to the depth of the lesion, is classified as superficial (i.e., capillary), deep (i.e., cavernous), and compound (i.e., capillary cavernous) hemangioma [2]. Histologically, hemangioma is the proliferation of endothelial cells that can manifest at any parts of the body and appears mostly in the head and neck area [4, 9]. Intraoral hemangioma appears in the head and neck area unfrequently, but oral mucosa and skin are the most affected tissues in the oral cavity followed by the surrounding bone and muscle. Hemangioma is characterized by three stages: (a) proliferating, (b) involuting phase, and (c) involuted phase. Proliferating phase (an age of 0–1 year) exhibits characteristics of rapid growth and multi-laminated basement membrane under endothelium. In involuting phase (an age of 1–5 years), on the other hand, proliferation of endothelial cells and apoptosis increase. Involuted phase (an age over 5 years) indicates the complete regression of lesion and is featured by histological fibrosis and fat deposition [10].

Vascular malformations are benign lesions which are caused by structural anomalies of vessels. Like hemangioma, these lesions can arise anywhere in the body and can be found at birth, in infancy, and in adulthood. Most commonly affected intraoral tissues are anterior two thirds of the tongues, palate, gingiva, and buccal mucosa. Vascular malformations can be categorized as low-flow (i.e., capillary, venous, lymphatic), high-flow (i.e., arterial), and combination lesions (i.e., arteriovenous, lymphatico-venous, capillary-lymphatico-venous). In contrast to hemangioma, histologically, they do not reveal endothelial proliferation and do not multiply or regress throughout life [10].

The diagnosis of these benign vascular lesions requires clinical examination, biopsy, sonography, computed tomography (CT), magnetic resonance imaging (MRI), and angiography. The goals of treatment are as follows:

1. Prevent the complications that affect the patient’s well-being.
2. Not to cause permanent physical deformities.
3. Reduce the stress of patients and family from esthetic problems.
4. Better to avoid treatments which leave scars.
5. To reduce infection and pain, ulcer should be treated appropriately [6].

Treatment options for small and peripheral vascular lesions are conventional surgical excision, laser therapy, cryotherapy, selective embolization, sclerotherapy, and medical treatment using beta blocker or steroid.

When the lesions are larger and deeper, embolization or obliteration to adjacent vessel is required. These treatments lead to irreversible tissue injury and gradual fibrosis. Currently, surgical resection is considered as the best treatment for vascular benign lesions which occurred in the head and neck. Surgical resection should be performed to prevent recurrence. However, surgical resection is limited when complete resection is not possible, when it may cause critical bleeding problem or when crucial organs can be injured. Extensive surgery in the oral cavity can induce a problem of chewing or swallowing.

For lesions that are small or located where esthetical conservation is required, sclerotherapy can be an alternative to surgical treatment [7]. Sclerotherapy can regress lesions partially or entirely and is effective for relieving symptoms. Furthermore, the procedure is simple, less invasive, and inexpensive. By using sclerotherapy, it is possible to receive treatment as an outpatient [3, 4]. However, sclerotherapy should be performed with care, because it can cause complications such as pulmonary embolism, anaphylaxis, nerve damage, increased pain, and disseminated intravascular coagulation [3].

For sclerotherapy, ethanol, sodium morrhuate, STS, and bleomycin can be used. STS is a sclerosant which has been used widely for the treatment of low-flow vascular lesion since 1940s. This acts on a lipid molecule of endothelial cells and induces surface injury and collagen exposure, causing inflammatory reaction and organization of thrombus [8]. As a result, injected vein undergo fibrosis, the vessel lumen obliterate partially or completely, and the lesions regress [3, 4, 9]. STS should be injected minimally at interval of 5–7 days between each injections [5].

Kim et al. reported three cases that were treated by injecting 1 or 3 % STS in benign oral vascular lesions [11]. They injected 2 mL of 3 % STS in the hemangioma of the lower labial mucosa, 2 mL of 1 % STS in the hemangioma of the right buccal mucosa, and 2 mL of 3 % STS in the venous malformation of the palate. The result of treatments was satisfied esthetically and functionally. Min et al. reported two cases that were treated by injecting 1 % STS in the venous malformation of the tongue and left buccal mucosa [5]. After the injection, the lesions were regressed remarkably. Alakailly et al. reported 13 cases that were treated by injecting 3 % STS in the venous malformation of the head and neck [12]. They injected a minimum of 0.5–2 mL into each lesion. The volume was depending on a ratio of 0.5 mL for each 2 cm of lesion size or a quarter volume of the lesion. Four patients had complete, five patients had 75 %, two patients had 50 %, and one patient had 25 % shrinkage of the lesion size. But, one patient had no response. After the injection, two patients had a superficial ulceration which healed without leaving scar. One patient had mild ecchymosis.

Same for other sclerosing agent, STS occasionally can cause pain, ulceration, edema, cardiovascular collapsing, and anaphylaxis [8]. In these cases, to reduce the side effects, 1 % STS was injected into the lesions instead of 3 % STS. In all cases, the size of lesions was remarkably regressed without complications after the injection. Because the reactions to sclerosant agent are different for each patient, there is no clear guideline for the standard therapeutic dose of STS for the treatment of benign oral vascular lesions. According to the manufacturer’s instructions, 0.5–2.0 mL of 3 % STS can possibly inject into a lesion per one dose and it must not be administered over 2 mL into the lesion.

## Conclusions

STS is effective in treating benign vascular lesions of small size in oral cavity. Advantages of STS are that it is inexpensive and it makes possibility to avoid the risk of surgery. However, as it is not the definitive treatment that can completely remove the lesion, it is effective when the size of lesion is small, and the growth of lesion is slow. Therefore, in order to use STS, patient selection and evaluation of patient is important and thorough understanding of its side effects is necessary to prevent complications.
